# Cytomegalovirus: clinical features and management

**Published:** 2020-03-30

**Authors:** Jonel Steffen, James Rice

**Affiliations:** 1 Vitreoretinal and uveitis consultant: University of Cape Town, Cape Town, South Africa.; 2 Vitreoretinal consultant: University of Cape Town, Cape Town, South Africa.

**CMV retinitis is the most common cause of vision loss in patients with acquired immunodeficiency syndrome (AIDS). Early recognition and management by a multidisciplinary team are essential.**

Cytomegalovirus (CMV) retinitis is the most common opportunistic infection of the eye, usually occurring in HIV-positive patients with CD4 counts <50 cells/µl.

## Clinical features

Patients with CMV retinitis present with unilateral or bilateral visual loss and/or floaters, without any pain. There is usually minimal or no vitritis and a clear view of the retina.

There are three clinical forms of CMV retinitis:

1. **Fulminant:** There are dense, white, well-demarcated areas of retinal necrosis with retinal haemorrhages, often described as a “pizza pie” appearance (Figure 1a). It tends to occur along the vascular arcades and over weeks gradually extends along the vessels in a ‘bushfire-like’ pattern. It may also affect the optic nerve head.
2. **Indolent:** Mild, granular opacification of the retina with very few retinal haemorrhages, which starts in the retinal periphery and progresses slowly.
3. **Frosted branch angiitis:** The least common form, in which perivascular exudation is the most obvious feature (Figure 1b).

## Diagnosis

The diagnosis is usually made clinically by dilated fundoscopy. The most common differential diagnoses include necrotising herpetic retinitis caused by herpes simplex (HSV) or varicella zoster virus (VZV), as well as toxoplasmosis gondii and syphilis. If the clinical diagnosis is uncertain, then syphilis serology and an aqueous or vitreous sample sent for polymerase chain reaction (PCR) for CMV, HSV, VZV and *Toxoplasma gondii* is helpful. If HIV status is unknown, then perform HIV serology, CD4 count and viral load. CMV retinitis is an AIDS-defining illness in HIV positive patients.

**“Patients with CMV retinitis should be managed by a multidisciplinary team including an ophthalmologist and a physician or infectious disease specialist.”**

## Treatment

Patients with CMV retinitis should be managed by a multidisciplinary team including an ophthalmologist and a physician or infectious disease specialist. Anti-CMV treatment (intravitreal and/or systemic) is given by the ophthalmologist, whilst the physician or infectious disease specialist should start antiretroviral treatment and help with monitoring and treatment of any side-effects of the medication.

### Systemic anti-CMV treatment

Oral valganciclovir (induction dose 900 mg twice-daily for 14–21 days, followed by maintenance dose of 900 mg daily) is the CMV treatment of choice because of its ease of administration. The disadvantage is that monitoring for bone marrow suppression and renal toxicity is required routinely to detect these adverse effects. It is also expensive and not available in all centres.

**Figure 1a:**
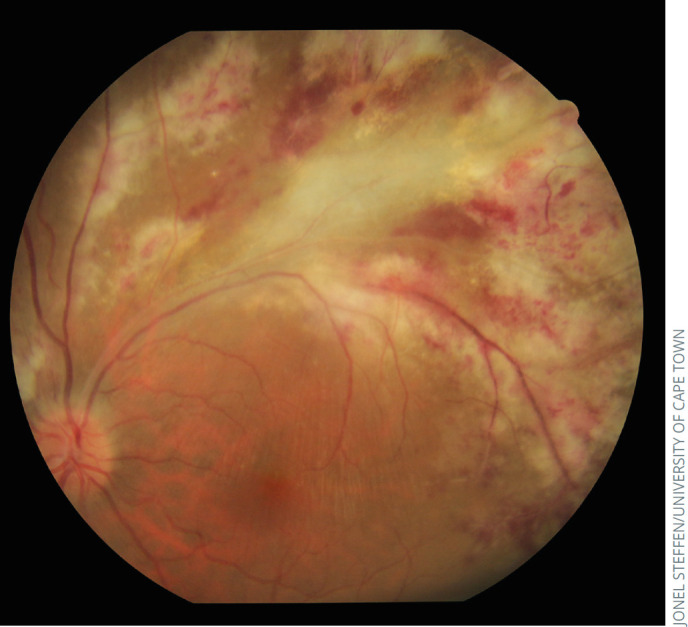
Fulminant CMV retinitis

**Figure 1b:**
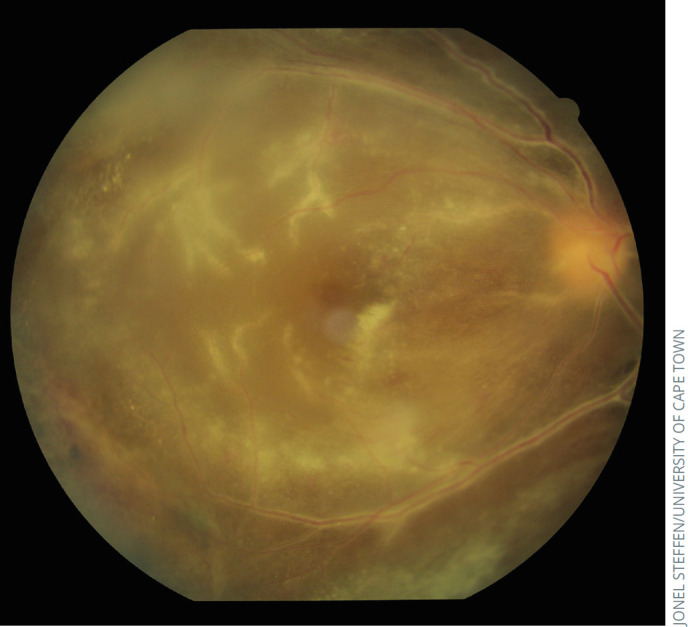
Frosted branch angiitis

Intravenous ganciclovir (induction dose 5 mg/kg every 12 hours for 14–21 days, followed by maintenance dose of 5 mg/kg/day) can be used as an alternative, but this requires inpatient treatment for the intravenous therapy. All patients who have sight-threatening CMV retinitis (infection within 1 disc diameter of the fovea or optic disc) should also receive weekly intravitreal ganciclovir injections for the first two weeks (see below for details).

**Figure 2:**
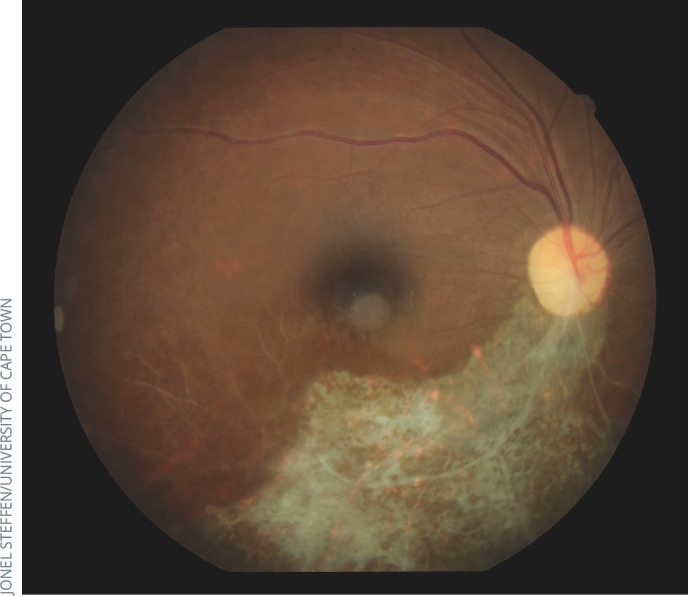
Inactive CMV retinitis

**Figure:**
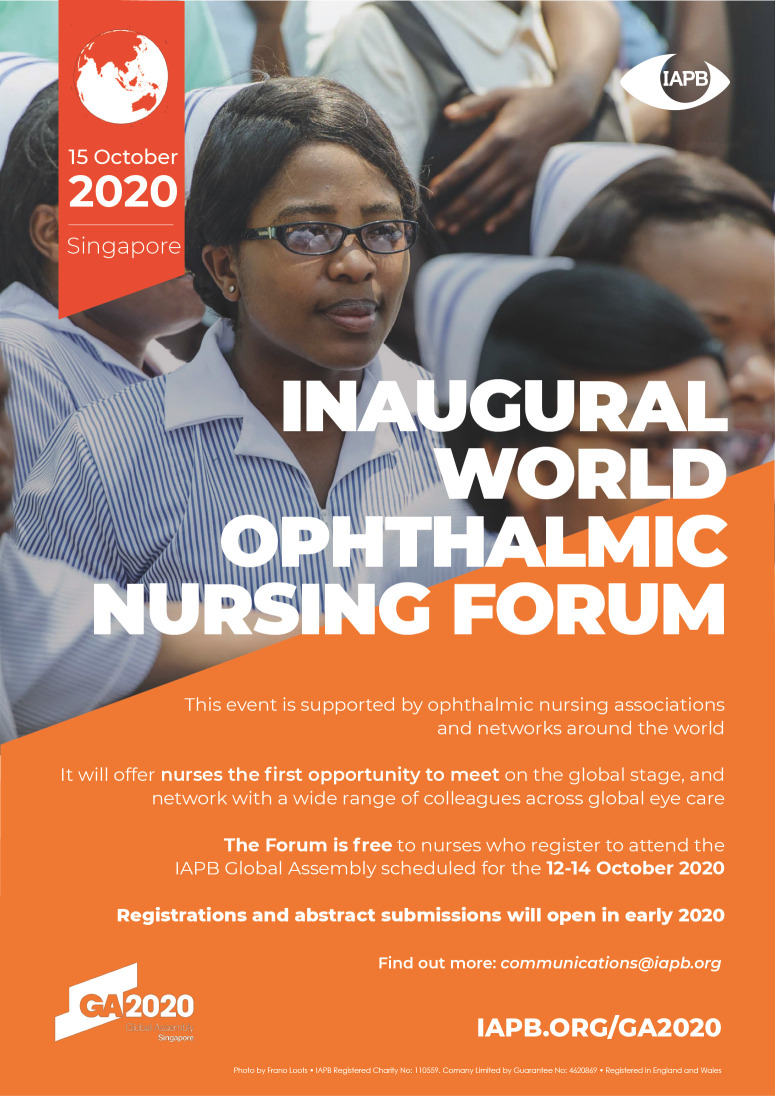


### Intravitreal anti-CMV treatment

Weekly injections of intravitreal ganciclovir (2.5 mg in 0.1 ml) into the affected eye/s is the treatment of choice in many resource-limited units. It is inexpensive and can be given as an outpatient treatment. The disadvantages are that it does not protect the fellow eye or treat systemic CMV infection, requires trained and experienced clinicians, and carries the low – but potentially sight-threatening –risks associated with intravitreal injections, such as endophthalmitis.

### Antiretroviral treatment (ART)

Ideally, ART should be started two weeks after starting CMV treatment to reduce the risk of immune recovery uveitis, but it may be more appropriate to start both at the same time in resource-limited settings.

A complete discussion of all the treatment options is beyond the scope of this article and can be found at UpToDate.^1^

## Follow-up and complications

We advise the use of fundus photographs to monitor treatment response. Initially, patients should be assessed weekly. Inactive CMV lesions will stay the same size, become less opacified and retinal haemorrhages will resolve (Figure 2).

CMV scars have areas of thin, necrotic retina which may form holes and lead to rhegmatogenous retinal detachments; these require pars plana vitrectomy and silicone oil tamponade.

CMV treatment (intravitreal or systemic) may be stopped when all the following criteria are met^1^:

1. CMV retinitis is completely inactive.
2. Patient is on ART with CD4 count of > 100 cells/µl or CD4 count that has increased by 50 cells/µl above baseline.
3. Patient has received at least three months of CMV treatment.

At our unit, we sometimes stop intravitreal injections before three months if criteria 1 and 2 are met, and we then monitor for any recurrences at one, three and seven weeks before discharging the patient.
